# Pharmacokinetic pharmacodynamic modeling of analgesics and sedatives in children

**DOI:** 10.1111/pan.14712

**Published:** 2023-06-21

**Authors:** Maddlie Bardol, Shan Pan, Suellen M. Walker, Joseph F. Standing, Joy M. Dawes

**Affiliations:** ^1^ Infection, Immunity and Inflammation, Great Ormond Street Institute of Child Health University College London London UK; ^2^ Department of Anaesthesia and Pain Medicine Great Ormond St Hospital NHS Foundation Trust London UK; ^3^ Developmental Neurosciences Program, UCL Great Ormond St Institute of Child Health University College London London UK; ^4^ Department of Pharmacy Great Ormond St Hospital NHS Foundation Trust London UK

**Keywords:** children, pain, pharmacodynamic, pharmacokinetic, sedation

## Abstract

Pharmacokinetic pharmacodynamic modeling is an important tool which uses statistical methodology to provide a better understanding of the relationship between concentration and effect of drugs such as analgesics and sedatives. Pharmacokinetic pharmacodynamic models also describe between‐subject variability that allows identification of subgroups and dose adjustment for optimal pain management in individual patients. This approach is particularly useful in the pediatric population, where most drugs have received limited evaluation and dosing is extrapolated from adult practice. In children, the covariates of weight and age are used to describe size‐ and maturation‐related changes in pharmacokinetics. It is important to consider both size and maturation in order to develop an accurate model and determine the optimal dose for different age groups. An adequate assessment of analgesic and sedative effect using pain scales or brain activity measures is essential to build reliable pharmacokinetic pharmacodynamic models. This is often challenging in children due to the multidimensional nature of pain and the limited sensitivity and specificity of some measurement tools. This review provides a summary of the pharmacokinetic and pharmacodynamic methodology used to describe the dose–concentration–effect relationship of analgesics and sedation in children, with a focus on the different pharmacodynamic endpoints and the challenges of pharmacodynamic modeling.

## 1 INTRODUCTION

Childhood pain ranges from acute to chronic, and includes procedural, disease‐related, break‐through and other types of pain. Undertreated, unrecognized or poorly managed pain in childhood leads to important and long‐lasting negative consequences that continue into adulthood.^1^ The adequate assessment and effective management of pain and sedation is an important part of the care given to children in hospital, and several validated tools and monitors exists to guide therapy.

Analgesic requirements are influenced by age‐related changes in both pharmacokinetic (PK) and pharmacodynamic (PD) response. Pharmacokinetic pharmacodynamic (PKPD) modeling is a tool which has informed analgesic, sedative and anesthetic target‐controlled infusion (TCI) dosing regimens and universal PKPD models for a number of drugs have now been described over a wide age range.^2^, ^3^, ^4^, ^5^ In order to develop these models, the target‐concentration effect relationship needs to be well‐defined in order to determine a target concentration associated with a target effect. Drug PK parameters can then be simulated to determine optimal dosing regimens for specific populations.^6^, ^7^, ^8^, ^9^

A major barrier to designing PKPD studies in children is the lack of PD scoring systems to help delineate the target effect. In this review we will discuss PKPD modeling in analgo‐sedation, with a particular focus on PD endpoints and the difficulties associated with these measures in children.

## 2 MAIN ARTICLE

### 2.1 Pain, nociception and sedation

Effective management of pain in children is necessary to reduce distress and minimize acute physiological instability. However, due to its subjective and multidimensional nature, pain may be inconsistently assessed and inadequately treated. Regular standardized pain assessment is necessary for titration of analgesia to individual needs and assessment of treatment response.

Nociception, in contrast to pain, is not a subjective feeling but the physiological response to nociceptive stimuli and by itself is very difficult to measure in the clinical environment. Monitoring nociception may allow a reduction in stress response beyond simply controlling hemodynamics and may identify patients with a higher likelihood of severe pain.

Sedation can be described as a continuum of levels of consciousness, ranging from conscious to deep sedation, and finally to general anesthesia.^10^ Effective sedation requires identifying appropriate goals (e.g., managing anxiety, modifying behavior/movement that hampers care, and minimizing physical discomfort), titration according to patient need, regular monitoring and techniques appropriate for the clinical context and practice setting (e.g., brief procedure or prolonged intervention; ward or intensive care; mechanical or spontaneous ventilation).^10^

### 2.2 Pain measurement tools

Pain measurement tools are available for children across a broad age range and should be matched to the age and development of the child.^11^ Over 65 pediatric pain scales are published, examples of which are listed in Table 1. Many of these tools have been validated for use in specific populations, for example, premature infants.

**TABLE 1:** Pediatric pain and sedation assessment tools (adapted from Schug et al.^11^).

Scale	Indicators	Score	Utility
Neonates			
Premature Infant Pain Profile (PIPP)	Facial expression, cry, breathing patterns, arms, legs, state of arousal	Each scored on 4‐point scale (0,1,2,3) <6 = minimal pain >12 = moderate to severe pain	Procedural pain in preterm and term neonates Postoperative pain in term neonates
Neonatal Pain, Agitation and Sedation Scale (N‐PASS)	Crying/irritability, behavioral state, facial expression, extremities tone, vital signs (heart rate, blood pressure, oxygen saturation)	Each scored on 5‐point scale (−2, −1, 0, 1, 2); total score: −10 to +10 with minus scores reflecting responses if sedated	23–40 weeks Postoperative pain Procedural pain Persistent pain Sedation level
COMFORTneo	Alertness, calmness/agitation, respiratory response (ventilated) or crying (spontaneous ventilation), body movement, facial tension, muscle tone	Each scored on 5‐point scale (1–6); total score: 6–30. Score >14 indicating moderate–severe pain/distress	24–42 weeks Prolonged pain Sedation
Infants and children: self‐report tools			
Faces Pain Scale–Revised (FPS‐R)	6 graphically depicted faces Simplified version for 4 years Electronic versions available	Neutral anchors Verbal anchors: No pain to very much pain	>5/6 years Acute pain Postoperative pain Chronic pain
Numeric Rating Scale (NRS)	Pain intensity from 0 to 10 Electronic version	0 = No pain or hurt 10 = Worst pain or hurt you can imagine/possible	>6 h Acute pain Postoperative pain Chronic pain
Infants and children: composite scales			
Face, Legs, Activity, Cry, Consolability (FLACC)	Face, legs, activity, cry, consolability	Each scored on a 3‐point scale (0, 1, 2); total score 8–40	Young children Postoperative pain
COMFORT	Alertness, calmness/agitation, respiratory response, physical movement	Total score 8–40	Newborn to adolescent Distress in pediatric intensive care unit
COMFORT B (behavioral elements)	Muscle tone, facial expression, mean arterial pressure, heart rate		Postoperative pain 0–3 years Downs syndrome 0–3 years Burns 0–5 h Postcardiac surgery in term infants
Sedation and agitation scales			
Ramsey Sedation Scale (RSS)	Six categories of awareness Uses auditory and tactile stimuli	Score from 1 (awake, anxious and agitated) to 6 (deeply asleep, unrousable to any stimuli)	Sedation for pediatric procedures
Richmond Agitation Sedation Scale (RASS)	Ten levels of sedation/agitation: five of sedation, one of calm alertness, and four levels of agitation	Score from −5 (unrousable) to +4 (combative)	Sedation monitoring on pediatric intensive care unit

Self‐report is considered the “gold standard” for pain assessment but is not feasible for all ages or practice settings. Observer‐based behavioral measurement tools can be used in younger and preverbal children and specific measures are available for children with cognitive impairment. A wide variety of pain assessment tools have been used in PKPD modeling, including N‐PASS,^12^ COMFORT^13^, ^14^ and faces scales.^15^ Commonly used sedation scales include the Ramsey Sedation Score (RSS) and the Richmond Agitation Sedation Scale (RASS) (Table 1). The RSS is frequently used in PKPD modeling studies for sedative drugs.^16^

Ideally, all researchers would use the same pain scales, selected from a limited number of externally and widely used scales. Data from different trials could then be compared with each other and pooled for meta‐analysis. The Pediatric Initiative on Methods, Measurement, and Pain Assessment in Clinical Trials (PedIMMPACT) statement, published in 2008, aimed to address these issues by recommending six core outcome domains for acute pediatric pain trials and the use of specific pain scales in children of different ages and clinical scenarios.^17^ However, a large number of pain scales continue to be used in pediatric pain studies.^18^

Several innovations in pain assessment are ongoing, for example, the development of the standardization of patient‐reported outcomes through the patient‐reported outcomes Measurement Information System (PROMIS)^19^ with work underway to validate the PROMIS measures in children in a variety of painful conditions. Ongoing technological innovations, such as the use of electronic daily diaries in pediatric pain trials for real‐time measurement of pain and will profoundly affect research and clinical practice.^1^

### 2.3 Nociception monitors

The most frequently used response to “surgical stress” is an increase in sympathetic activity or the corresponding decrease in parasympathetic tone. Changes in cardiac autonomic control (i.e., increased heart rate), increased peripheral vasoconstriction, pupillary dilatation and an increase in galvanic skin conductance offer a relatively easy access to the evaluation to such sympathetic response. In addition to these “simple” reactions, stress may also influence salivary cortisol levels, heart rate variability, electroencephalographic (EEG) and electromyographic (EMG) patterns, and the threshold of peripheral reflexes. The most commercially available monitors are based on the detection of one, two, or multi‐parameters,^20^ examples of which are given in Table 2. Although these nociception monitors appear to reflect intraoperative stimuli better than traditionally used parameters such as blood pressure or heart rate, to date, none have shown a convincing and clinically relevant benefit to support their routine use. There are also challenges in using these monitors in the very young. A neonate will display behavioral responses to distress when awake but hemodynamic parameters and motor reflexes under anesthesia are poor indicators of anesthetic depth.^21^

**TABLE 2:** Examples of nociception monitors (adapted from Ledowski et al.^20^).

Device	Measurement	Score	Utility
Single‐parameter scores			
Analgesia nociception index (ANI)	Cardiac parasympathetic tone	0–100; dimensionless	ANI (>50) has a high negative predictive value for the absence of acute postoperative pain and pain in the ICU
Skin conductance	Peripheral (skin) sympathetic tone	Number of fluctuations in skin conductance per second (NFSC) (*n*)	Many confounders in awake subjects NFSC >0.2 may correlate with severe nociception and significant postoperative pain More useful as a “smoke detector” in case of significant intraoperative stress response
Pupillometry	Sympathetic tone (pupillary innervation)	Absolute or changes in pupillary width (mm %^−1^)	Influenced by depth of anesthesia, opioids Pupillometry‐guided analgesia may reduce opioid consumption, persistent pain, responsiveness to postoperative opioids
Nociceptive flexion reflex (NFR) threshold (NFTS Paintracker)	RIII reflex modification by level of analgesia	mA	May aid prediction of response to noxious stimuli Limited predictive value for postoperative pain
Two‐parameter scores			
Surgical plethysmographic index (SPI)	Peripheral vascular and cardiac sympathetic tone	0–100; dimensionless	Lower opioid consumption and shorter times to tracheal extubation Ideal intraoperative score may be closer to 30 (vs. frequently quoted <50) Guidelines for ideal range not well validated Not recommended in awake patients
qNOX	EMG/EEG patterns associated with nociception	0–99; dimensionless	May help predict probability of sudden movement in response to stimulation
Multi‐parameter scores			
Nociception level (NOL) index	Four parameters: skin galvanic response, plethysmographic pulse wave, temperature and accelerometry	0–100; dimensionless	May discriminate noxious stimuli slightly better than standard hemodynamics Ideal intraoperative NOL may be 10–25

Nociceptive cortical activity can also be studied using techniques such as near‐infrared spectroscopy (NIRS) and functional magnetic resonance imaging. These techniques provide an indirect measure of neuronal activation inferred from changes in cerebral blood flow in response to a stimulus.

Tools measuring pain intensity have been used in PKPD modeling to describe the change in drug response over time. Clinically required procedures such as blood tests, which have an expected intensity, frequency, and duration of stimulus, are often used to ensure a precise estimation of PKPD parameters and improve the robustness of the model.^22^

### 2.4 Cerebral function monitors

Cerebral function monitors offer a more objective method of monitoring sedation, particularly in the ICU setting. The processed EEG is useful in monitoring sedation in children, however, specific interpretation of these signals is required in neonates and infants. Maturation of the neonatal brain, in particular neuronal myelination, results in age‐dependent changes in the EEG frequency bands and their relative amplitude (power). Common indices include spectral edge frequency (SEF), density spectral array, burst suppression ratio, Patient State Index and Narcotrend Index.^21^ However, depth of anesthesia monitoring is of little value in neonates and infants where the target concentration is seldom known and EEG monitoring is inadequate.^23^

Quantitative EEG is the most commonly used biomarker for anesthetic drug activity in both preclinical and clinical PKPD modeling.^4^, ^24^, ^25^, ^26^, ^27^ Because such mechanism‐based PKPD models characterize processes on the causal path between drug administration and effect, they have important advantages in terms of translation and prediction. The cortical EEG signals reflect voltage fluctuations within neurons in the brain and can be analyzed using different indices such as Bispectral index (BIS) and entropy, depending on the algorithm used. BIS provides a score between 0 and 100, inversely correlated with the level of sedation and results from an amalgamation of time, frequency and spectral analysis.^28^ Entropy evaluates the depth of hypnosis through analysis of the EEG signal's irregularity. Wave patterns become more regular as anesthesia deepens, and are processed into two indicators: state entropy (SE): 0–91 and response entropy (RE): 0–100, with different characteristics in frequency bandwidths and temporal window length.^28^ BIS is the most common tool used as a PD endpoint to evaluate the efficacy of sedatives.^4^, ^26^, ^27^ However, it is important to note that EEG dynamics change throughout childhood due to the expression of cortical and subcortical network developmental processes. All infants lack the frontal alpha predominance and coherence seen under sevoflurane anesthesia in adults, and theta and alpha oscillations appear at around 4 months of age.^29^ Therefore, EEG monitoring‐based algorithms relying on the frequency components of the EEG need to be adjusted to account for age‐related changes in the EEG, specifically in infants.

NIRS, NIRS combined with EEG, and MRI can also be used to measure brain activity evoked by noxious stimulation, modulation by analgesia, and effects of sedation/anesthesia.^30^, ^31^, ^32^ The identification of robust and developmentally‐sensitive pain indicators should continue to be a priority, so that better tools are available for use in both clinical practice and research.^1^ These specialized techniques may provide additional outcomes for future PKPD studies.

### 2.5 Pharmacodynamic endpoints

PD endpoints measure a drug's activity in the body using biomarkers and/or clinical outcomes to quantify efficacy and safety. Various measures of drug response are used routinely in clinical practice to guide therapy, however PD endpoints are often parameterized (e.g., turned into a score) in order to compare treatments, particularly in the research setting.^33^ Examples of validated age‐appropriate PD‐related scoring systems include the COMFORT‐B score used to measure sedation in pediatric critical care. However, there is no single preferred validated scoring system able to reliably detect the varying degrees of anesthesia in pediatric patients. Depth of anesthesia is a theoretical construct without an accepted standard for clinical measurement, which is a limitation in many PD modeling studies.^28^

The relationship between drug exposure and PD endpoint has been inadequately studied in children. Developmental (ontogenetic) changes can affect how a drug is handled by the body^33^ and this variability in drug exposure affects both the desired pharmacological response and the risk of adverse effects. Although there is increasing knowledge of PK in neonates and children, there is a paucity of information related to PD in children and addressing this pediatric PD knowledge gap must be the focus of future research.

### 2.6 Pharmacological treatment

The evidence‐base for the pharmacological management of acute pain in children is increasing but there are still relatively few controlled trials in neonates. Many sedatives and analgesics are prescribed with limited evaluation in children.

Acetaminophen is a frequently used analgesic, either alone for mild pain or as part of combination therapy for more severe pain. Data for safe and effective dosing is now available.^34^, ^35^, ^36^ Nonsteroidal anti‐inflammatory drugs also provide benefit alone or as part of multimodal analgesia if there are no contra‐indications to use.^34^, ^35^

Opioids remain the first‐line treatment for children with moderate to severe postoperative pain.^37^ Titrated bolus dosing can be used for procedural pain in intensive care but the long‐term risk–benefit of opioids for sedation in mechanically ventilated neonates is debated.^38^ In children, PKPD models have described the optimal doses of intraoperative remifentanil and fentanyl,^5^, ^39^ postoperative oxycodone^7^ and morphine.^8^, ^13^ However, there is a clear need for additional PKPD data to inform opioid use in children of different ages and clinical settings. Pharmacogenetic differences may also influence efficacy or safety of opioids. For example, ultrarapid metabolism of codeine and tramadol by the enzyme CYP2D6 has been associated with respiratory depression and death, and led to recommendations to avoid these drugs in children under 12 years and older children at increased risk (e.g., following tonsillectomy, obstructive sleep apnea, obesity).^37^

Benzodiazepines, such as midazolam have previously been the sedative agent of choice. In addition to sedation and anxiolysis, anterograde amnesia with midazolam may provide benefit, particularly on PICU.^40^ However midazolam may also be associated with paradoxical agitation, tolerance, dependence, and withdrawal, and associations with impaired neurodevelopmental outcome have reduced its use for prolonged sedation on NICU.^40^, ^41^ The use of alpha‐2‐adrenergic receptor agonists such as clonidine and dexmedetomidine has increased, due to the lower incidence of adverse effects (particularly respiratory depression and withdrawal), agitation and delirium and potential reduction in stress response.

Propofol and combinations of opioid/propofol or ketamine/propofol(ketofol) can be used for deeper sedation,^42^ but careful titration and adequate monitoring is essential to ensure safety. Eleveld et al. have validated a PKPD model to predict propofol concentrations and BIS for a broad population range.^4^, ^43^

### 2.7 Modeling approaches

Due to age‐dependent differences in PK (e.g., clearance, metabolism, distribution) and PD (physiological and behavioral response, susceptibility to side effects), using linear weight‐based extrapolation of doses from adults does not usually provide equivalent analgesia or sedation in children of all ages. Modeling approaches for analgesics and sedatives have increased to identify the adequate dose regimen for different age groups, improve pharmaceutical study design, and optimize drug administration protocols.

A PKPD approach can be used to develop mathematical models that describe and predict the PK and PD of analgesic and sedative agents, and link PK to PD. However, PKPD relationships have not been fully explored for all agents. The PKPD models generate parameter estimates such as maximum analgesic effect, the concentration of half‐maximum effect and the effect‐site equilibration rate constant. They also describe the between‐subject variability of these parameters which allows dose adaptation for special populations by identification of subgroups (e.g., responders vs. nonresponders).^22^ As sedatives are often used in combination with analgesics, it is important to test the influence of these co‐medications by including them in the PKPD efficacy models.^24^, ^39^

### 2.8 Pharmacokinetic models

The population approach used in PKPD modeling uses a statistical method called mixed effects to fit the models (find optimal values of model parameter estimates). Mixed effects, or multilevel modeling, allows repeated measures within individuals to be grouped by allowing model parameters to vary between individuals, and is the generally accepted optimal way to analyze population PKPD data.^44^ Fitting a model allows the estimation of PK parameters such as volume of distribution and clearance from observed data (such as plasma concentration) given dosing‐specific variables (i.e., dose, time) and patient‐specific covariates (e.g., body weight). The most popular method for analyzing population PK data is nonlinear mixed‐effects (NLME) modeling. With the NLME approach, the model is fitted to data from all individuals simultaneously to estimate both population average PK parameters and variability. There are two different sources of variability: between‐subject variability (BSV) which is the variance of parameters between subjects, and the unexplained variability called residual unexplained variability (RUV) that may arise from measurement noise or model misspecification. In order to avoid bias, mixed effect modeling includes the correlation of data points within individuals and the variability of parameters between individuals. This population model fitting generates PK population parameters (including their between‐subject variability) and the variance of variability unexplained by the model.^44^ PKPD data are mostly analyzed using the compartmental approach, which uses hypothetical compartments connected to each other to represent the human body^45^ (see Figure 3). The central compartment consists of the plasma and tissues, and following an intravenous bolus, the drug distribution is practically instantaneous. For drugs administered via extravascular routes (such as buccal midazolam or intranasal dexmedetomidine), absorption to the central compartment is usually described by a first‐order kinetics. The peripheral compartments consist of tissues where the drug distribution is slower compared to the central compartment. However, for most of the models, these compartments have no real anatomical meaning, they are treated as systems with lumped organs or tissues, for example, for simplicity the whole human body may be seen as a single compartment during PK modeling.

#### 2.8.1 Allometry and maturation function

In pediatric population modeling, the model must account for changes in size and age due to the growth and development of organs over time. For children older than 2 years, a scaling based only on size describes the PK parameters adequately. However for younger infants, maturation of glomerular filtration and enzymes involved in drug metabolism should be taken into account since organs such as the liver and kidneys are still in development during the first months of gestation^44^, ^46^ and generally are completed within the first 2 months of postnatal life. A standard method of scaling for size and maturation is to fix the allometric weight exponent to 0.75 and use a sigmoidal maturation function driven by PMA to estimate the fractional decrease in allometrically scaled clearance with decreasing age^46^:

(1) $$CL_{i}=CL_{T}\cdot {\left(\frac{WT_{i}}{70}\right)}^{0.75}\cdot \frac{\mathrm{PM}A_{i}^{\text{Hill}}}{\mathrm{PM}A_{50}^{\text{Hill}}+\mathrm{PM}A_{i}^{\text{Hill}}}$$

where, CL is drug clearance in an individual, $CL_{T}$ is the typical CL for a 70 kg adult, WT is body weight, $\mathrm{PM}A_{50}$ is the PMA (usually in weeks) for CL to reach 50% mature, and Hill is the shape parameter.

However, there are other methods of scaling for size and the different types of maturation functions that have been used. Germovsek et al.^47^ compared the fit of all the major types of published model for size and age scaling of clearance in children and found that no model gave superior fit to this standard model describe above. The volume parameters scale linearly with body weight, so the allometric weight exponent is 1. A good example of this method is the allometric model developed by Rigby‐Jones et al.^48^ to describe the PK of remifentanil in children. It has been shown that this model extrapolated adequately to adults whereas other PK models that did not use allometry scaling extrapolated poorly in children particularly in neonates.^5^ Indeed, in the remifentanil PK model developed by Eleveld et al.,^5^ one of the volumes of distribution deviated from the linear scaling with body sizes and showed a decrease for larger body sizes. For drugs with slower maturation times, such as propofol and dexmedetomidine, the addition of a maturation function is even more important to account for the maturational changes in the neonatal period and infancy. Whether to fix or estimate allometric exponents is an issue of much debate^49^; however, most authors follow the considerable body of biological prior information and fix the exponent to 0.75, thereby also saving a degree of freedom in model fitting.^44^

In the analgesia and sedation literature, several PKPD modeling papers include both children and adults. In order to precisely estimate the parameters for different age populations, these models include allometric scaling.^2^, ^5^, ^27^ Using allometric scaling and maturation function on the PK parameters is a useful way of comparing model parameter estimates across studies.^50^, ^51^ These techniques have been discussed in a number of recent PKPD modeling reviews.^21^, ^52^, ^53^

### 2.9 Pharmacodynamic models

There is no standard method to develop a PKPD model. Either PK and PD can be estimated simultaneously (“simultaneous” method), or the PK model is built first, then the PD is estimated with the PK parameters fixed if the model is unstable (“sequential” method).^54^ The simultaneous method is considered the “gold standard”; however, in numerous cases the model is not stable enough to estimate the parameters using this method, so the sequential method is preferred.^54^

There are different ways to model the PD variables depending on whether the variables are continuous or categorical. For *continuous* PD variables such as visual analogue pain scales or EEG index signal (BIS and SE), linear, log linear and *E*_max_ models have been used.^24^ Often a delay between observed concentration and effect occurs, in which case effect compartment models and indirect response models are suitable.^55^ For *categorical* PD variables, the data are analyzed with logistic regression. A model of cumulative probabilities can be used to describe ordinal categorical data such as the COMFORT scale. However, Peeters et al^14^ in their PKPD study of propofol in children modeled the COMFORT score as a continuous variable with an *E*_max_ value corresponding to the maximum score of the scale.

Most of the PKPD models evaluate the relationship between efficacy and drug concentration. However, some models also evaluate the relationship between concentration and safety parameters such as blood pressure and heart rate.^56^ Unlike PK models, PD scaling for pediatric patients, including allometric scaling and maturation over age, is not universally applied. However, developmental PD differences should be considered for inclusion in the model when feasible, especially in infants and neonates. For example, BIS and entropy monitors are less linearly correlated in infants and there is an age dependent effect on the pharmacodynamic relationship.^57^, ^58^ Sciusco et al. applied a mixed effects model population approach with concurrent BIS and entropy monitoring comparing model fit characteristics of the concentration‐response relationship at different ages in pediatric surgery.^28^ They could not exclude the possibility of a maturational effect of the pharmacological model itself (i.e., nonsigmoidal relationship) so used the measure of absolute conditional individual weighted residuals (CIWRES), a metric of model goodness‐of‐fit which should be independent of covariate effects because it is based on individual model predicted values. With rich data they were able to explain that the observed trend was due to differences in PD endpoint, that is, the data produced by the EEG monitors.^28^

Few PKPD models have included the influence of age on parameters using either allometry scaling or linear relationship.^24^, ^27^ Some models have also included other covariates (e.g., infusion rate,^24^ weight^59^ and mechanical ventilation^13^) in order to obtain a better estimation of the parameters. In certain situations the inclusion of the metabolite concentration also improves the model fit and therefore should be taken into account in the PKPD model building^59^ For instance, the model published by Garrido et al^59^ showed that using the concentration of the tramadol metabolite was more relevant when describing the relationship between the drug and some efficacy endpoints.

#### 2.9.1 Models for continuous pharmacodynamic variables

Continuous variables are numeric variables that can take on any score or value within a measurement scale. There are two main types of continuous variables, interval and ratio. Interval variables have numerical values which can be ordered and the distance between each score is equal and static (e.g., temperature). If this variable has a clear 0 point which indicates that there is none of that variable and the ratio of the scores make sense, this variable is called ratio (e.g., weight, age, distance). For instance, if respondents were being surveyed about their pain levels on a numerical rating scale of 0–10, a respondent with a pain level of 10 is assumed to have twice the pain experienced as a respondent who selected a pain level of 5.

##### Direct models

First, it is important to define the PD model which describes the response data. The direct models are the most commonly used PD models in the literature due to their simplistic but also mechanistic nature.^55^ In these models, a change in blood drug concentration causes an effect which can be observed instantaneously. These models can be linear and log linear which suppose a proportional relationship between concentration or log‐transformed concentration and effect as described by the following equations^55^:

(2) $$\begin{array}{c} E=m\cdot C_{p}+E_{0}\\ E=m\cdot \log\left(C_{p}\right)+E_{0} \end{array}$$

where E is the effect, $C_{p}$ is drug plasma concentration, $E_{0}$ is the baseline effect, and m is the slope.

**FIGURE 1:**
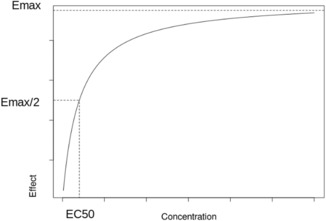
Plot of an $E_{\max}$ model of drug concentration versus effect; $E_{\max}$ is the maximum effect and $EC_{50}$ is the drug concentration for 50% *E*_max_ effect observed.

The linear model may provide a good description of concentration‐effect for small concentration ranges. However, the log linear model is more appropriate for concentrations that produce effects between 20% and 80% of the maximum effect (*E*_max_). These models have some disadvantages since they are not able to predict a saturated *E*_max_ for high concentrations. This issue is removed in the *E*_max_ model. The limited resources of biological systems (e.g., receptor protein) are taken into account in this model by the notion of maximum effect represented by the plateau in Figure 1.^60^ When the effect is between 20% and 80%, the relationship between concentration and effect is log linear. The slope of this log linear function can be controlled by adding an exponent to the *E*_max_ model; this model is called sigmoidal *E*_max_ model:

(3) $$E=E_{0}+\frac{E_{\max}\cdot C_{p}^{n}}{EC_{50}^{n}\cdot C_{p}^{n}}$$

where E is the observed effect, $E_{\max}$ the maximum effect, $EC_{50}$ is the drug concentration for 50% *E*_max_ effect observed, $E_{0}$ the baseline effect, $C_{p}$ the drug concentration, and *n* the slope exponent.

**FIGURE 2:**
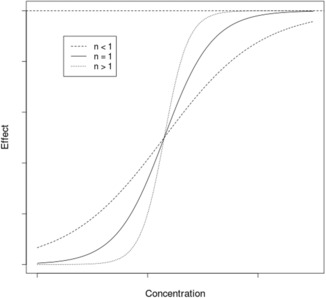
Plot of a sigmoidal *E*_max_ model with a logarithmic abscissa and three different values of *n*. *n* refers to the slope factor (Hill factor) and measures sensitivity of the response to the dose range of the drug, determining the steepness of the dose–response curve. The dotted line represents the *E*_max_.

When *n* > 1, the hyperbolic function becomes sigmoidal hyperbolic. However, in PKPD this *n* parameter has no real physiological meaning. For *n* < 1, *E*_max_/2 is reached quickly then the evolution to *E*_max_ is slow whereas for *n* > 1, $E_{\max}$ is reached more rapidly with lower concentration (Figure 2).

It is common to observe a time delay between a dose given and the observed effect. In this case the PK and PD do not belong to the same compartment. The relationship between effect and concentration is represented by a hysteresis, which is the typical curve observed in indirect response models. Hysteresis describes the time delay between plasma concentration and effect and can be caused by different physiological mechanisms such as sensitization or formation of active metabolites.^61^

##### Effect compartment models

**FIGURE 3:**
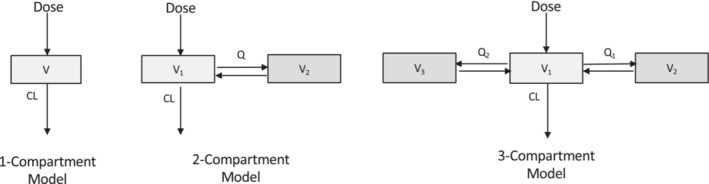
Schematic diagram of compartmental PK models; each rectangle represents a compartment, V and V_1_ represent the central compartment, V_2_ and V_3_ represent the peripheral compartments. Q and CL represent the inter‐compartmental and elimination clearances, respectively.

In the case of hysteresis, the PKPD model most often used is the biophase model, also called the effect compartment model. The concept is to integrate a hypothetical effect compartment to the PK compartment models as described in Figure 3. It is assumed that the amount of drug in the effect compartment is negligible and therefore the mass balance in the system is not affected. By adding the hypothetical effect compartment, the drug concentration in the effect compartment is then linked with observed PD effect.

$K_{e0}$ is the effect‐site elimination rate constant and determines the time delay between observed plasma drug concentration and the PD effect. The *K*_e0_ can also be parameterized as a half‐time *K*_e0_ (ln (2)/*K*_e0_), which is directly correlated with the time of equilibration between the central compartment and the effect site. In an extreme case where $K_{e0}$ is estimated as a very large value, this would suggest a fast equilibrium between plasma and effect compartments and therefore the effect compartment can be removed without a delay in effect. In case of a delay in effect, the effect compartment can then be regarded as a direct model making it possible to use direct PD models such as an $E_{\max}$ sigmoid model to describe the effect.

The biophase model is the most commonly used PKPD model for analgesics and sedatives.^4^, ^5^, ^26^, ^27^ For instance, several studies used an effect compartment to link concentration of propofol and sedation measured by BIS. Since the BIS value inversely correlates with the level of hypnosis, the relationship between the effect‐site concentration of propofol and BIS can be evaluated using an inhibitory sigmoid *E*_max_ model.^4^, ^25^, ^26^, ^61^ In certain situations, it is possible to include more than one PD effect compartment. For example, Blussé van Oud‐Alblas et al^26^ used an inhibitory sigmoid *E*_max_ model with two effect compartments to describe the relationship between propofol concentration and sedative effect.

##### Indirect response models

The indirect response corresponds to the PD response caused by the drug which alters the production ($K_{\text{in}}$) or dissipation ($K_{\text{out}}$) process of endogenous factors. Although this model is frequently used in PKPD modeling for adult and pediatric populations, it assumes the measurement and inclusion of a biomarker, which is rarely used to assess pain and sedation. Hence, there is no indirect model published to date describing the effect of analgesics and sedatives in children.

#### 2.9.2 Models for categorical pharmacodynamic variables

A categorical variable is a measurement scale composed of a set of finite number of categories or distinct groups which can be nominal or ordinal. A nominal variable does not have an intrinsic order and is called binary or dichotomous if it has only two possible outcomes such as sex or awake versus asleep. However, in clinical practice, variables are commonly rank ordered, as in observational pain scores such as COMFORT. In this instance, the data is described as ordinal categorical data.

Although observational pain scores are often divided into different sub‐scores or items, only the overall score is used in most models as a PD endpoint. However, different combinations of sub‐scores can give the same overall score. In order to evaluate the contribution of each sub‐score to the PD response, the item response theory (IRT) approach can be used, although it requires very rich extensive data. This approach combined with PK modeling can improve the effectiveness and precision of PKPD analysis.^62^

In PD modeling, it is sometimes helpful to model discrete data as if it were continuous, as illustrated by Sheiner using bromfenac data.^63^ For example, when handling categorical or ordinal data, such as pain scores, that are measured on a discrete scale but reflect a continuous underlying variable. By treating the data as continuous, standard statistical models can be used, such as linear regression or mixed effect models to determine the dose–response relationship.

##### Analysis of binary data

The analysis of binary endpoints involves logistic regression. The two categories can be quantified as “success” (*S*) and “failure” (*F*). The response *R* is denoted by 1 for S and 0 for F. The corresponding proportion or probability *P* has a Bernoulli distribution as described in the following equations^55^:

(4) $$\begin{array}{c} P\left(S\right)=\pi \\ P\left(F\right)=1-\pi \\ P\left(R=r\right)=\pi^{r}{\left(1-\pi \right)}^{1-r} \end{array}$$

Because of the binary nature of the data, π can take values only between 0 and 1. To ensure this, it is necessary to transform p from a −∞ to +∞ which is done using the logistic transformation or logit. The logit transforms the 0 to 1 probability scale to a −∞ to +∞ scale and is expressed as a function of π ^55^:

(5) $$\text{logit}\left(\pi_{i}\right)=\ln\left(\frac{\pi_{i}}{1-\pi_{i}}\right)$$

In PKPD modeling, many strategies can be adopted to evaluate the influence of the PK (exposure) and covariates on this response. These variables must be implemented on transformed scales, as is shown is the following example^55^:

(6) $$\begin{array}{c} \text{Logit}\left(\pi_{i}\left(t_{j}\right)\right)=\theta_{1}+\theta_{2}C_{e}\left(t_{j}\right)+\eta_{i}\\ P_{j}=\frac{e^{\text{Logi}t_{j}}}{1+e^{\text{Logi}t_{j}}} \end{array}$$

where $C_{e}\left(t\right)$ represents the drug concentration at effect‐site at time point *t* for the jth patient j which can be linear or follow an $E_{\max}$ model,^13^ thetas are the Logit baseline, η the residual error for the observation which is independent and follows a normal distribution with mean 0 and variance $\omega^{2}$ and P is the predicted probability of success S in the jth patient.

This model has been used to describe a binary sedation score (1 = awake, 2 = drowsy/asleep) after administration of midazolam in children, and the addition of midazolam metabolite was found to improve the model fit of this logistic regression PKPD model.^64^ More recently, Valkenburg et al^13^ developed a logistic model in children to describe the sedation effect of morphine. In this model, the COMFORT‐B score was used as categorical binary data to assess sedation (COMFORT‐B score < 11 corresponding to oversedation).

##### Analysis of ordinal categorical data

The handling of categorical data with multiple categories poses challenges, particularly with interpretation and graphical representation. To analyze ordinal categorical data, a model of cumulative probabilities can be used since the response can take different values Y of probability $P\left(Y\right)$. In this model, it is assumed that a response Y+1 corresponds to symptoms “stronger” than category Y. If we consider a trichotomous variable Y=1,2 or 3 the probabilities can be modeled as following:

(7) $$\begin{array}{c} P\left(Y=1\right)=1-P\left(Y>1\right)\\ P\left(Y=2\right)=P\left(Y>1\right)-P\left(Y>2\right)\\ P\left(Y=3\right)=P\left(Y>2\right) \end{array}$$

If necessary, it is possible to merge original categories into a lesser number of categories, especially when a small number of observations are available for each category. For this model, the probability also needs to be transformed in logit function:

(8) $$P\left(Y>=1\right)=\frac{e^{\text{Logit}\left(P>=1\right)}}{1+e^{\text{Logit}\left(P>=1\right)}}$$

The most common way to model such data is to use a proportional odds model.

(9) $$\text{Logit}\left[P\left(Y>=j\right)\right]=\alpha_{j}+\mathrm{\beta x}$$

where α represents the logit baseline probability for each category ($\alpha_{1}<\alpha_{2}<\ldots \alpha_{j}$), β is the drug effect parameter identical for all categories such as *E*_max_ and EC_50_ and x is the predictor vector which can be dose, drug exposure or any other variables.

Cumulative probabilities were used by Garrido et al^59^ to model the efficacy of intravenous tramadol in children. In this study, the effect was evaluated using ordinal categorical scores to describe the child comportment, each score corresponding to a crying status (0 = not crying, 1 = crying but consolable, 2 = crying but inconsolable). Response variables were treated as ordinal categorical data and analyzed using logistic regression. Goodness of fit plots were used to represent mean model predicted and mean raw data cumulative probabilities versus time. The visual predictive check was used to explore the model. Scores from 1000 datasets were simulated. For each simulated dataset and time of the measurement, the mean probability corresponding to each score was computed. Then, for each measurement time and score, the overall median value was calculated. Finally, the overall median values were plotted against the mean raw data probabilities.

## 3 CONCLUSION

Effective and safe management of pain and sedation is important for children of all ages for humanitarian reasons and to minimize distress, and may improve both acute and long‐term outcomes.^65^ Increased validation and standardization of pain assessment tools will improve clinical practice. A range of validated tools are available for use in different clinical settings, and additional measures such as changes in stress hormones and measures of cortical activity have been utilized in the field of research. Quantifying nociception/analgesia remains the ultimate goal in order to reduce severe postoperative pain and also the incidence of opioid related side effects.

Population PK modeling uses mixed effects to investigate variability in drug responses in individuals. The main sources of variability in children are age (maturation) and weight (size). An understanding of concentration‐response relationships helps guide dosing to achieve a target concentration that achieves a target effect. Pharmacokinetic modeling of analgesic and sedative drugs in children has greatly increased over the last few decades, however, there is still a huge lack of PD studies. The major barrier to future PKPD studies is the lack of validated PD scoring systems.

Different PD models are used depending on the type of response data. Linear, log linear or *E*_max_ models can be used to model continuous data, and logistic regression for categorical data. The use of logit is acceptable when it encompasses 0 or 1 but the handling of categorical data with multiple categories requires careful consideration and expertise in PD modeling.

## FUNDING INFORMATION

MB, SP and JMD received no specific external funding. SMW is supported by Great Ormond Street Hospital Children's Charity (Grant W1071H). JFS receives support from the Medical Research Council (Grant 1002305).

## REFLECTIVE QUESTIONS

1. Which measurement tools are commonly used as PD endpoints in neonates, infants and children?
2. In pediatric PK modeling, how do we account for changes in size and organ maturation of the child?
3. How can categorical data be handled in PD modeling?

## CONFLICT OF INTEREST STATEMENT

The authors report no financial conflict of interest relevant to this manuscript. Suellen Walker is a Section Editor for *Pediatric Anesthesia*.

## Data Availability

Data sharing is not applicable to this article as no new data were created or analyzed in this study.
